# The Micrococcus of Soft Chancre

**Published:** 1886-10

**Authors:** 


### *THE MICROCOCCUS OF SOFT CHANCRE.*

Dr. R. DeLuca, after a series of experiments upon this subject, has arrived at the following conclusion:

1. The contagious principle of soft chancres resides in a specific micro-organism, the *micrococcus ulceris*. This may be isolated and cultivated, and when inoculated in man, reproduces the soft chancre.

2. Besides the micrococcus ulceris in the soft chancre, are found several other micro-organisms, among which the most important are the *staphylococcus aureus* and *citreus* and the *streptococcus pyogenus*.

3. These last named micrococci are the cause of the simple bubo accompanying soft chancre.

4. When the micrococcus ulceris reaches the lymphatic glands with these bacteria, the ulcerating or phagedenic buboes are produced.

In this case the pus of the bubo assumes the characteristics of the pus of the ulcer only after a lapse of from twenty-four to forty-eight hours, because the micrococcus ulceris requires the presence of air for its growth, and only develops after the lapse of twenty-four hours.

